# Loss of a Clueless-dGRASP complex results in ER stress and blocks Integrin exit from the perinuclear endoplasmic reticulum in *Drosophila* larval muscle

**DOI:** 10.1242/bio.201511551

**Published:** 2015-04-10

**Authors:** Zong-Heng Wang, Catherine Rabouille, Erika R. Geisbrecht

**Affiliations:** 1 Division of Cell Biology and Biophysics, School of Biological Sciences, University of Missouri, Kansas City, MO 64110, USA; 2 Hubrecht Institute-KNAW & University Medical Center Utrecht, 3584 CT Utrecht, The Netherlands; 3 The Department of Cell Biology, UMC Utrecht, 3584 CX Utrecht, The Netherlands; 4 Department of Biochemistry and Molecular Biophysics, Kansas State University, Manhattan, KS 66506, USA

**Keywords:** *Drosophila*, Muscle, Integrin, Clueless, dGRASP, Trafficking

## Abstract

*Drosophila* Clueless (Clu) and its conserved orthologs are known for their role in the prevention of mitochondrial clustering. Here, we uncover a new role for Clu in the delivery of integrin subunits in muscle tissue. In *clu* mutants, αPS2 integrin, but not βPS integrin, abnormally accumulates in a perinuclear endoplasmic reticulum (ER) subdomain, a site that mirrors the endogenous localization of Clu. Loss of components essential for mitochondrial distribution do not phenocopy the *clu* mutant αPS2 phenotype. Conversely, RNAi knockdown of the *Drosophila* Golgi reassembly and stacking protein GRASP55/65 (dGRASP) recapitulates *clu* defects, including the abnormal accumulation of αPS2 and larval locomotor activity. Both Clu and dGRASP proteins physically interact and loss of Clu displaces dGRASP from ER exit sites, suggesting that Clu cooperates with dGRASP for the exit of αPS2 from a perinuclear subdomain in the ER. We also found that Clu and dGRASP loss of function leads to ER stress and that the stability of the ER exit site protein Sec16 is severely compromised in the *clu* mutants, thus explaining the ER accumulation of αPS2. Remarkably, exposure of *clu RNAi* larvae to chemical chaperones restores both αPS2 delivery and functional ER exit sites. We propose that Clu together with dGRASP prevents ER stress and therefore maintains Sec16 stability essential for the functional organization of perinuclear early secretory pathway. This, in turn, is essential for integrin subunit αPS2 ER exit in *Drosophila* larval myofibers.

## INTRODUCTION

Integrins are integral transmembrane heterodimers that mediate the adhesion of epithelial sheets with extracellular matrix components (ECM), such as laminin and fibronectin. This adhesion is essential for diverse biological processes including embryonic development, cell migration, and muscle attachment. Initiation and maintenance of these integrin adhesion complexes is highly regulated. In addition to basic transcriptional and translation control, integrins require transport to sites of adhesion and subsequent protein turnover in response to either ligand binding and/or modulation of intercellular signaling (Margadant et al., 2011; Rodriguez-Boulan et al., 2005). Determining the dynamic control of exo/endocytic integrin trafficking within various cell types is crucial to understanding morphogenesis and homeostasis in multicellular organisms.

Mammals display 18 α and 8 β subunits, so far known to comprise 24 distinct integrin heterodimers (Hynes, 2002), while *Drosophila* has only 3 α and 2 β Position Specific (PS) integrin chains (called αPS1, αPS2, αPS3, βPS and βν) that assemble into cell-type specific heterodimer complexes (Bulgakova et al., 2012). This subunit simplicity in the fly model exemplifies the utility of *Drosophila* as a model to understand integrin function in developmental processes and cell-ECM interactions. In both flies and vertebrate systems, integrin complexes accumulate at muscle attachment sites (MASs) and the costameres (Charvet et al., 2012; Schejter and Baylies, 2010; Schnorrer and Dickson, 2004; Schweitzer et al., 2010). While many studies in the *Drosophila* model have focused on the role of integrins in muscle attachment (Estrada et al., 2007; Gilsohn and Volk, 2010; Liu et al., 2013), little is known about trafficking of integrin subunits in the secretory pathway.

The majority of integrin anterograde trafficking studies has been conducted in cell culture and support a model whereby integrin dimers are transported via the canonical secretory pathway, likely mediated by interactions with other cytosolic proteins, including talin or calnexin (Lenter and Vestweber, 1994; Martel et al., 2000). The cytoplasmic domains of α/β subunits are necessary for efficient exit of some integrin dimers from the ER (Briesewitz et al., 1995; Ho and Springer, 1983). Talin can control the export of newly synthesized integrins in AT22 cells through binding to integrin cytoplasmic tails, possibly by exposing an export signal in the α integrin chain (Martel et al., 2000). Moreover, studies using conformation-specific monoclonal antibodies demonstrate that β1 integrins adopt an inactive, bent conformation after heterodimer formation with α subunits in the ER. This obligate dimer persists as transport continues through the Golgi to the plasma membrane (Tiwari et al., 2011).

*Drosophila* GRASP (dGRASP), the single ortholog of mammalian GRASP55/65, is one protein required for integrin subunit delivery. Originally characterized as peripheral Golgi proteins (Barr, 1997; Shorter, 1999), the GRASP family is required for a diverse array of processes, including the maintenance of Golgi architecture and unconventional protein secretion (Vinke et al., 2011). In the *Drosophila* follicular epithelium, integrin subunits are differentially transported to the basolateral surface of epithelial cells at the transition from stage (st.) 10A to st. 10B, when follicle cell remodeling is occurring. Specifically, the integrin α subunit (αPS1) that is expressed in these epithelial cells, gets retained in the ER upon loss of dGRASP (Schotman et al., 2008).

Membrane proteins, such as integrins, are sorted in the ER at ER exit sites (ERES), or transitional ER (tER) sites. Sec16 is a key player in maintaining the organization of ERES where it is thought to recruit coat proteins necessary for vesicle formation (Budnik and Stephens, 2009; Glick, 2014; Miller and Barlowe, 2010). In support of this, Sec16 protein localizes to budding cup-shaped structures on the ER in both human and *Drosophila* S2 cells (Hughes et al., 2009; Ivan et al., 2008), and loss of Sec16 function in yeast and metazoans results in a loss of ERES integrity and a block in protein secretion (Bhattacharyya and Glick, 2007; Connerly et al., 2005; Hughes et al., 2009; Ivan et al., 2008; Watson et al., 2006). Protein sorting at ERES is less understood. Many proteins get transported through COPII vesicles to the Golgi before reaching their final destination at the plasma membrane or outside the cell (Barlowe and Miller, 2013; Venditti et al., 2014), while an increasing body of literature describes alternative routes for protein delivery that bypass the Golgi (Rabouille et al., 2012).

Using the *Drosophila* musculature as a model to study integrin delivery, we focus on the Clu protein. Clu was originally identified in the prevention of mitochondrial clustering in *Saccharomyces*, *Dictyostelium*, *Arabidopsis*, and *Drosophila* (Cox and Spradling, 2009; Dimmer et al., 2002; Fields et al., 1998; Frederick and Shaw, 2007; Zhu et al., 1997). Clu has two predicted domains based upon primary sequence conservation with the human protein (KIAA0664); an undefined ‘Clu’ domain (residues 424–666) and a C-terminal tetratricopeptide repeat (TPR) domain, which may serve as a scaffold to mediate protein-protein interactions (Cox and Spradling, 2009). The only known protein that cooperates with *Drosophila* Clu and affects mitochondria is the E3 ubiquitin ligase Parkin (Cox and Spradling, 2009). Parkin ubiquitinates Mitofusin in the clearance of damaged mitochondria and loss of Parkin results in early onset Parkinson's disease (Guo, 2012). However, the role of Clu within the Parkin pathway and/or mitochondrial distribution are unknown.

Herein we unravel a novel, mitochondrial-independent role for Clu in the differential transport of integrin subunits in contractile muscles. Decreased levels of Clu lead to the retention of αPS2, which is phenocopied by loss of dGRASP function. Interestingly, loss of Clu and dGRASP leads to an increase in ER stress and a decrease in the number and size of ERES marked by Sec16 protein. We show that compounds alleviating this ER stress restore αPS2 export and ERES functional organization. Taken together, we propose that Clu together with dGRASP prevent ER stress to maintain Sec16 stability in the early secretory pathway and mediate αPS2 ER exit in *Drosophila* larval myofibers.

## MATERIALS AND METHODS

### Drosophila stocks and genetics

*Drosophila* stocks were maintained on standard cornmeal medium at 25°C, while RNA interference and rescue experiments were performed at 29°C. The original *clu^d087^* (*clu^d08713^*) and Clu:GFP protein trap line (CA06604) were provided by Rachel Cox (Cox and Spradling, 2009). *clu^ΔW^* was generated by imprecise excision of the *P[SUPor-P]CG8443^KG02346^* insertion. This deletion removes the 5′UTR and start codon of Clu as verified by PCR and sequencing. Unless noted, the *clu* mutants analyzed in our studies were *clu^d087^/clu^ΔW^*. Stocks obtained from the Bloomington Stock Center: *w^1118^* (BL-3605); *24B*-Gal4 (BL-1767) (LaBeau-DiMenna et al., 2012); *mef2*-Gal4 (BL-27390) (Geisbrecht et al., 2008); *daughterless (da)*-Gal4 (BL-55849) (Lindgren et al., 2008); *parkin^1^*/TM3 (Bl-34747) (Cha et al., 2005; Zhang et al., 2007); UAS-*YFP:Rab5* (BL-24616) (Zhang et al., 2007); UAS-*YFP:Rab7* (BL-23641); *spq*-Y*FP:KDEL-ER* (BL-7195) (LaJeunesse et al., 2004); *spq*-UAS-*YFP:Golgi* (BL-7193) (LaJeunesse et al., 2004); UAS-*dGRASP:GFP* (BL-8507); UAS-*sec16 RNAi* (on II; Catherine Rabouille); UAS-*sec16 RNAi* (on III; Catherine Rabouille); UAS-*Rab5.S43N* (BL-9772) (Entchev et al., 2000); UAS-*Xbp1.EGFP* (BL-39720) (Sone et al., 2013). RNAi lines obtained from the Vienna *Drosophila* RNAi Center: UAS-*dgrasp RNAi* (v22564); UAS-*clu RNAi* (v42136 recombined with v42138 to generate a 2× *UAS-clu RNAi* stock); UAS-*marf RNAi* (v105261) (Debattisti et al., 2014). Standard recombination was used to generate necessary stocks and verified by complementation or PCR.

### Molecular biology and antisera generation

The ORF of the full length *clu* cDNA (isoform A) was PCR amplified, cloned into the proper reading frame into the Gateway entry vector, and transferred into the UAS-myc destination vector using standard protocols (*Drosophila* GATEWAY™ cloning system, Invitrogen). The sequenced UAS-*Clu:Myc* construct was injected by Genetic Services, Inc. to obtain transgenic flies. To make the dGRASP Ab, a region of the *dgrasp cDNA* corresponding to nucleotides 601–942 was PCR amplified, cloned into pGEX-4T-3, expressed in *E. coli* to generate a GST-dGRASP (domain B) fusion protein and injected into rabbits.

### Immunofluorescent staining and imaging analysis

L3 larvae were live-dissected in HL3 (70 mM NaCl, 5 mM KCl, 20 mM MgCl_2_, 10 mM NaHCO_3_, 115 mM sucrose, and 5 mM Hepes, pH 7.2) or PBS and fixed in 4% formaldehyde. Primary antibodies used: guinea pig anti-Clu (1:2000) (Cox and Spradling, 2009); rabbit anti-dGRASP (1:400) (this study); rabbit anti-Sec16 (1:500) (Ivan et al., 2008); mouse anti-βPS-integrin [CF.6G11, 1:50, Developmental Studies Hybridoma Bank (DSHB)]; mouse anti-αPS-integrin (CF.2C7, 1:20, DSHB); rabbit anti-GFP (1:500, Invitrogen); mouse anti-ATP5 (15H4C4; 1:400, Mitosciences); mouse anti-BiP (1:100; Babraham Institute). Secondary antibodies used were Alexa Fluor 488 or Alexa Fluor 568 (1:400, Molecular Probes). Phalloidin 594 was used for F-actin labeling (Molecular Probes).

### Immunoprecipitation and western blots

Third instar larva were homogenized in lysis buffer (50 mM Tris-HCl pH = 7.5, 150 mM NaCl, 1 mM EDTA, 10% glycerol, 1% Triton X-100) mixed with 50 µg/ml PMSF, 1× Halt protease inhibitor cocktail (Pierce Biotechnology, Inc.). After centrifugation at 4°C at 12,000 ***g*** for 15 min, the supernatant for immunoprecipitation was incubated with 25 µl anti-Myc conjugated beads, 25 µl GFP-Trap beads (ChromoTek), for 4 h at 4°C. Beads were washed three times with lysis buffer and boiled in 5× Laemmli buffer. For protein level analysis, proteins were just extracted in lysis buffer. The protein samples were then separated by 6% SDS-PAGE, transferred to polyvinyl difluoride membranes (Pierce Biotechnology, Inc.), and probed with primary antibodies: mouse anti-Myc (9E10, 1:1000, Sigma), rabbit anti-GFP (1:500, Invitrogen), rabbit anti-Clu (1:1000) (Goh et al., 2013), rabbit anti-dGRASP (1:2000), rabbit anti-Sec16 (1:2,000), or mouse anti-α-Tubulin (1:100,000, B-512, Sigma), followed by incubation with Horseradish Peroxidase (HRP) conjugated secondary antibodies (1:5000, GE Healthcare) and detection using the ECL Plus Western Blotting detection system (Pierce).

### Fluorescence *in situ* hybridization

FISH on larval muscles was performed as previously described (Gardiol and St Johnston, 2014). Plasmids obtained from BDGP were linearized for antisense or sense probes as follows: *αPS2 LP16423:* antisense EcoRI/Sp6, sense XhoI/T7; *βPS RE55238*: antisense XhoI/T3, sense BamHI/T7. Probes were transcribed using corresponding RNA polymerases (New England Biolabs). Larva dissected in HL3 buffer were fixed in 4% formaldehyde, washed, washed in PBS Tween 0.1%, pre-hybridized for 2 h at 55°C, and hybridized overnight with 2 mg of probe. Fillets then were washed and incubated with Alkaline Phosphatase (AP) conjugated anti-Dig (1:200, Roche) overnight. Probed mRNA was detected by HNPP fluorescent detection kit (Roche) followed by fluorescence secondary antibody staining.

### Lethality analysis

Flies of the appropriate genotype were placed in cages supplied with yeast paste on apple juice agar plates for egg laying. 100 embryos of the indicated genotype were transferred to a fresh apple juice agar plates and the number of viable animals at different developmental stages was recorded each day. After the eclosion of the first adult, the remaining pupae were kept for an additional 4 days to determine if any would eclose.

### Larval locomotion assays

Staged L3 larva of the indicated genotype were placed on a fresh apple juice agar plate for 15 min to acclimate to their surroundings. Mobility was video-recorded (640×480 pixel resolution) for 1 min. The videos were transformed into time-lapse images (200 frame/min). The Grid plugin of ImageJ was utilized to overlay lines on the time-lapse images (area per point = 150 pixels^2^) and the number of grids which larva crawled through was recorded and converted to mm/sec.

### Confocal imaging and statistics

Fluorescent images were collected on an Olympus Fluoview300 or Zeiss700 confocal systems with single z = 0.5 µm, 5–6 µm total for 20×; z = 0.4 µm, 4–5 µm total for 40×; and z = 0.35 µm, 3–4 µm total for 63× objectives, respectively. Maximum intensity projections of confocal z-stacks were processed by using ImageJ software (NIH). Montage images were obtained from continuous sub-z-stacks beneath the sarcolemma. All images were assembled into figures using Adobe Photoshop. Colocalization analysis was performed on multiple single step images after montage generation. The colocalization efficiencies were obtained by using JACoP plugin in ImageJ with Manders' Coefficient algorithm. For quantification for protein levels from Western blots, the band intensities were measured by ImageJ and normalized by the levels of both α-Tubulin from the corresponding genotypes and the same proteins in WT. Line profiles of fluorescence intensity were plotted as shown previously (Bothe et al., 2014; Folker et al., 2014). Single plane of confocal z-stack picture was opened in ImageJ. A line selection was made across the puncta or nuclei of interest. The fluorescence intensities of single or double channel(s) on the selected line were depicted with using “line profile” Macro.

## RESULTS

### αPS2 accumulates around the nuclei in clu mutants

**Fig. 1.**
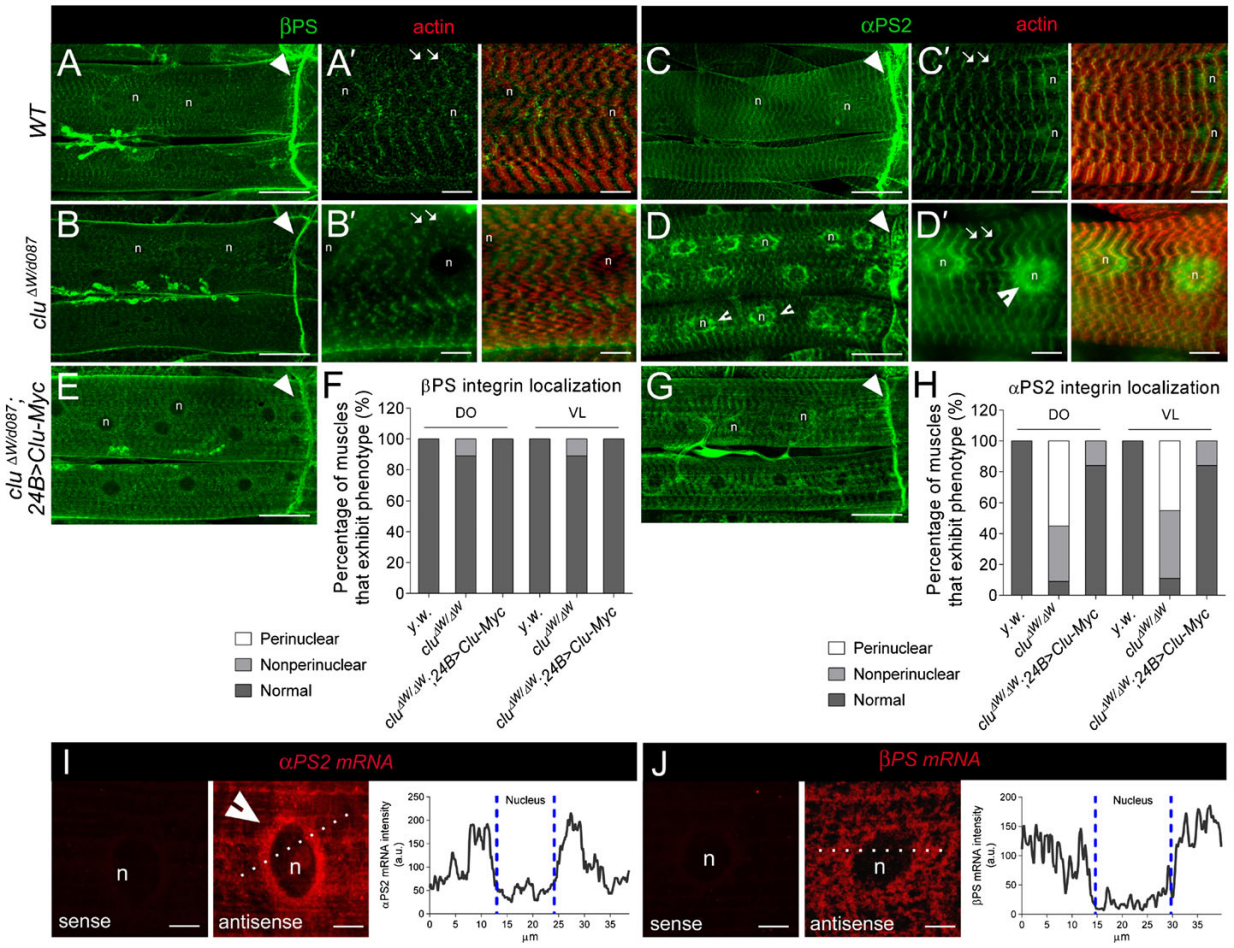
αPS2 accumulates within contractile muscles upon loss of Clu. (A–E,G) Immunolocalization of integrin proteins (green) and F-actin (red; phalloidin) in muscles of filleted L3 individuals (n = nucleus). (A–B′) In both WT and *clu*^−*/*−^ muscles, βPS integrin is found at MASs (arrowheads in A,B) and costamere structures that encircle the sarcolemma along the length of the muscle (arrows in A′,B′). (C,C′) αPS2 integrin also accumulates at the ends of WT muscles (arrowhead in C) and at costameres (arrows in C′). (D,D′) In *clu* mutants, the αPS2 subunit accumulates around the periphery of the nucleus, as indicated by the indented arrowheads. (E,G) The reintroduction of full-length *clu* cDNA into *clu* mutant muscle tissue has no effect on βPS integrin distribution (E) and restores the accumulation of αPS2 to its normal location within the cell (G). (F,H) Quantification of βPS and αPS2 integrin distribution in the dorsal oblique (DO; 16<n<36) and ventral longitudinal (VL; 16<n<36) L3 muscles of indicated the genotypes. (I,J) Fluorescent *in situ* hybridizations (FISH) in L3 muscle tissue. *αPS2* mRNA accumulates around the nuclei (I, middle panel; n = 42), while *βPS* mRNA appears evenly distributed throughout the muscle cell (J; n = 23). The sense probes for both mRNAs reveal little background signal (left panels). Quantitation of fluorescence intensity (dotted line) shows that the perinuclear signal of *αPS2* mRNA is higher than that of β*PS2* mRNA. Scale bars, 50 µm (A–E,G), 10 µm (A′–D′), 5 µm (I,J).

Clu was identified in a screen designed to identify new proteins in myogenesis. During our initial characterization of the *clu* gene (Z.-H.W. and E.R.G., unpublished), we immunostained *clu* mutants at different stages in development to look for defects in muscle development and/or maintenance. Upon staining for the integrin heterodimer complex, we made an interesting observation in the contractile musculature of *clu* mutants. As we reported previously by our group and others (LaBeau-DiMenna et al., 2012; Leptin et al., 1989; Nabel-Rosen et al., 1999), βPS (Fig. 1A,A′) and αPS2 (Fig. 1C,C′) normally accumulate at muscle attachment sites (arrowheads) and costameres (arrows) in contractile third instar larval (L3) muscles (arrows). In *clu* mutant L3 animals, βPS distribution appeared similar to WT L3 individuals (Fig. 1B,B′,F). However, loss of Clu resulted in an obvious accumulation of αPS2 protein in the region surrounding the muscle nuclei (Fig. 1D,D′), in a compartment that we call the perinuclear ER. This accumulation of αPS2 was strongly decreased upon the reintroduction of Clu protein into *clu* mutants (Fig. 1G,H). In addition to this increase in perinuclear staining, we occasionally observed a decrease in αPS2 levels at muscle attachment sites and costameres. This retention of αPS2 in *clu* mutants is consistent with a possible accumulation in the perinuclear ER.

### αPS2, but not βPS, is translated from a pool of targeted mRNA

To understand how αPS2 is retained in the perinuclear region of muscle cells, we hypothesized that αPS2 could normally be locally translated from a pool of targeted mRNAs around the nucleus followed by active transport to its final destination, as it is the case for Gurken in the *Drosophila* oocyte (Herpers and Rabouille, 2004). Conversely, *βPS* mRNA would be homogenously distributed. To test this, we performed RNA FISH for both integrin subunits to confirm our hypothesis. Although *αPS2* mRNA is present in the entire cell, it is concentrated around the nucleus (Fig. 1I, indented arrow), whereas *βPS* mRNA is homogenous and does not show this perinuclear concentration (Fig. 1J). This suggests that αPS2 is locally translated in the peripheral ER and might require Clu for its transport.

### Clu protein localizes to a subdomain of the ER

**Fig. 2.**
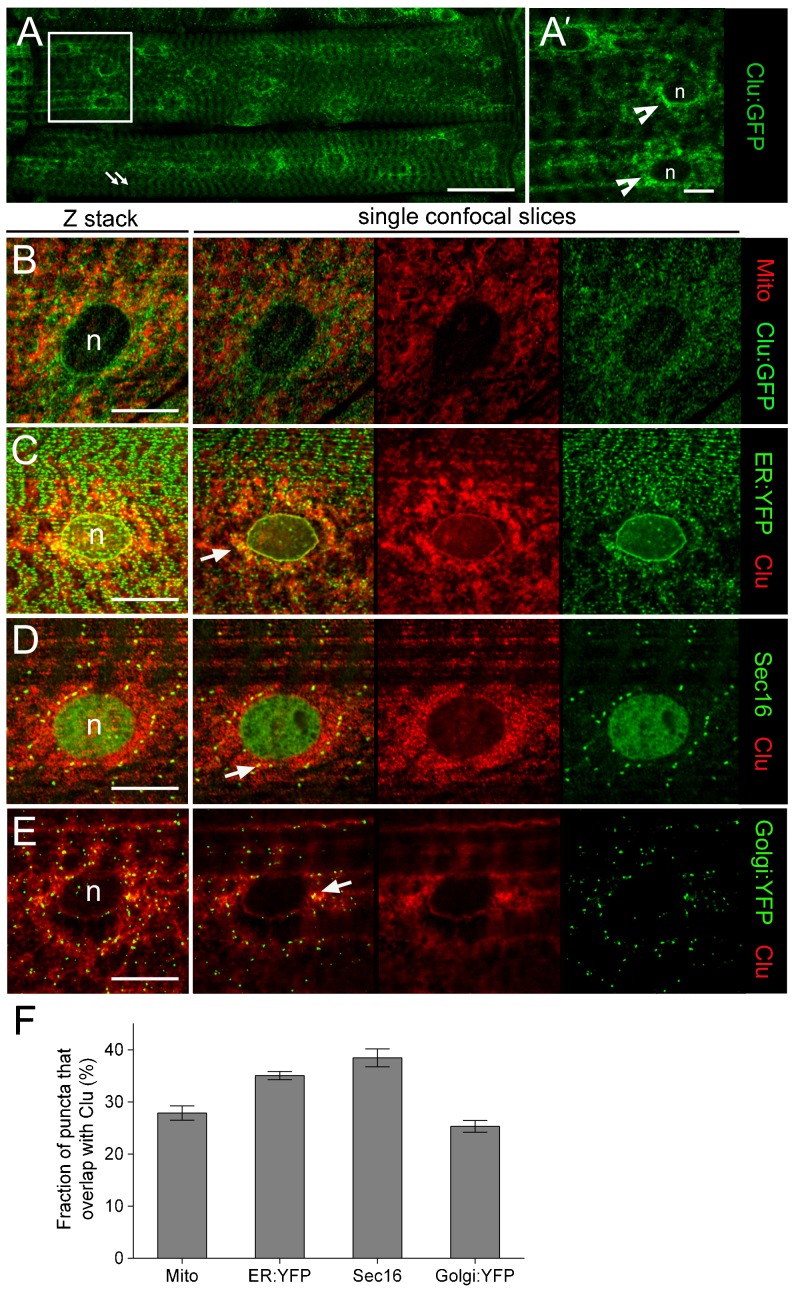
Clu localizes with dGRASP and Sec16 ER exit sites. (A–G) Muscle tissue from L3 larvae was dissected and immunostained to examine the subcellular localization of Clu protein. (A,A′) The Clu:GFP protein trap line (green) localizes in a repeated pattern within the muscle (arrows) and accumulates around the nuclei (n; indented arrows). The right panel is a close up of the boxed region in the left panel. (B) The perinuclear accumulation of Clu:GFP (green) reveals little colocalization with mitochondria (red; anti-Complex V). (C–F) An anti-Clu antibody (red) was used to confirm the Clu:GFP nuclear staining pattern and also to discern the localization of Clu puncta with other organelle markers (green) in WT larval muscle. A composite Z-stack is followed by representative single confocal slices. (C,D) Clu-positive puncta overlap with both a general ER marker (C) and the ERES protein Sec16 (D). (E,F) Clu colocalizes with a subset of Golgi:YFP puncta (E). (F) The percentage of puncta of each organelle marker that overlap with Clu protein. Colocalization was determined from multiple single plane images calculated using the Image J JACoP plug-in. Mean±s.e.m. Scale bars, 50 µm (A), 10 µm (A′,B–G).

**Fig. 6.**
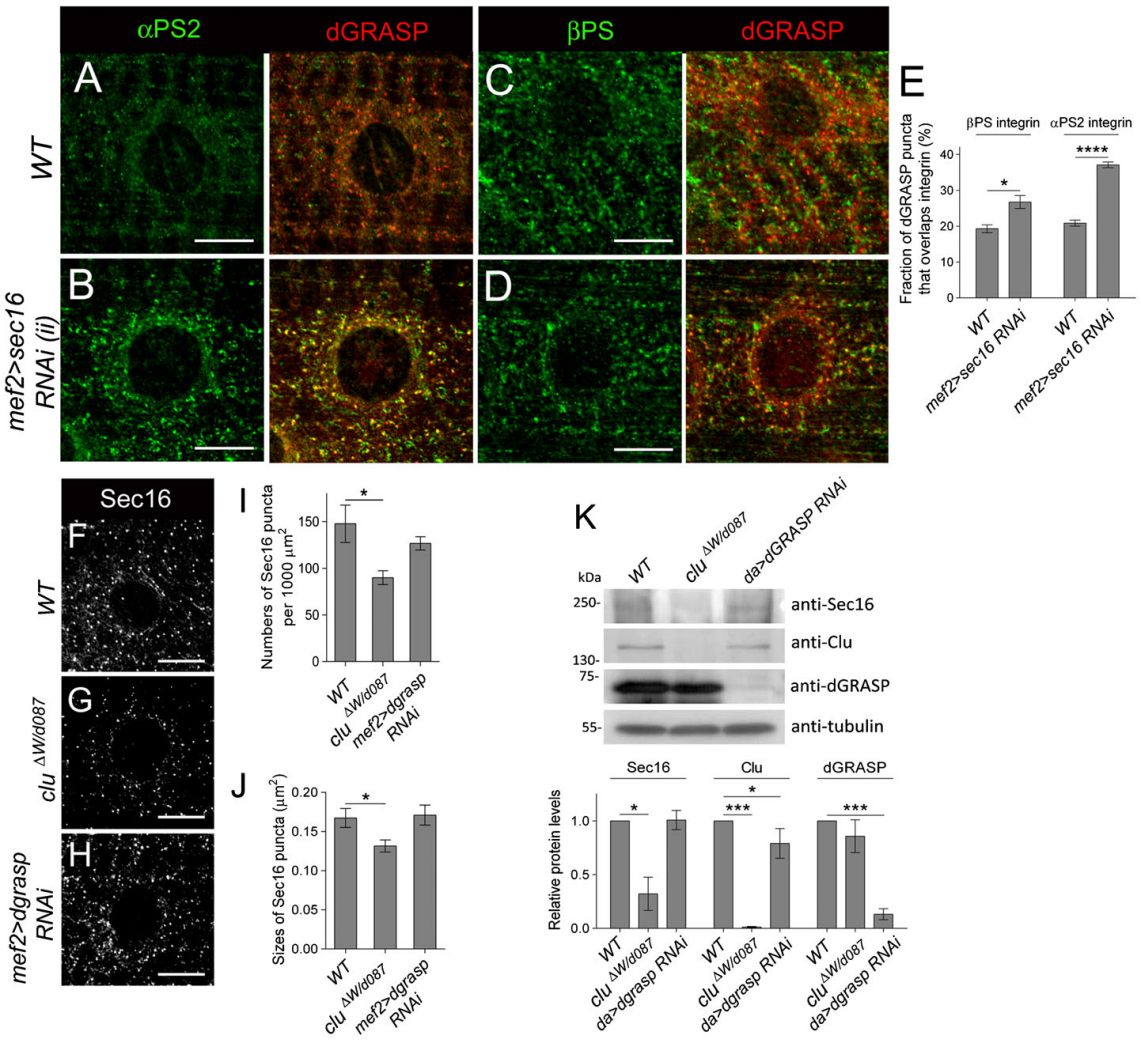
Sec16 protein levels are reduced in *clu* mutants. (A–D) Immunostaining of integrin (green) and dGRASP (red) in L3 contractile muscles. Low amounts of both αPS2 (A) and βPS (C) colocalize with dGRASP around nuclei (n) in WT muscle cells. (B,D) RNAi knockdown of Sec16 in muscle tissues results in the retention of αPS2 in dGRASP-positive puncta (B), while low levels of βPS accumulate around the nuclei (D). (E) Graph depicting the fraction of Sec16 puncta that overlap integrins based upon analysis of multiple images like those presented in panels A–F (*p<0.05; ***p<0.0005). (F–H) Perinuclear staining of Sec16 staining in the indicated genotypes. Sec16 puncta are reduced in *clu* mutants (G) when compared to WT (F) or *dgrasp*-depleted muscle tissue (H). (I,J) The number (I) and size (J) of Sec12-positive ERES are reduced in *clu* mutants. (K) Western blot and band intensity quantification of Sec16, Clu and dGRASP protein levels in the indicated genotypes. Sec16 protein levels are reduced in *clu*, but not *dGRASP* mutants (mean±s.e.m.; *p<0.05; ***p<0.005). Scale bars, 10 µm (A–D,F–H).

To better understand how Clu may affect αPS2 trafficking, we first focused on characterizing the location of endogenous Clu within the WT larval musculature. Analysis of the protein trap line *clu^CA06604^* revealed a broad distribution of Clu:GFP, including a faint but repeated pattern consistent with sarcomere organization (arrows), localized throughout the muscle cell (Fig. 2A). Remarkably, however, Clu protein (indented arrowheads) was found strongly concentrated around the nucleus in a pattern that mirrored the αPS2 perinuclear accumulation in *clu* mutants (Fig. 1D,D′). To ensure the GFP fusion tag did not interfere with its normal location within the myofiber, we verified the localization of the native Clu protein using an antibody generated against the N-terminal region of Clu (Cox and Spradling, 2009). The distribution of endogenous Clu (Fig. 2C,D) was identical to Clu-GFP (Fig. 2A), and the Clu protein staining appeared specific as Clu signals were reduced in *clu* mutants (Fig. 6K; supplementary material Fig. S1).

To further investigate the location of Clu within the muscle cell, we double labeled it with fluorescently labeled organelle markers followed by quantification of a region surrounding the nucleus to determine the percentage of overlap between these markers and Clu-positive signal. As Clu is in close proximity to mitochondria in *Drosophila* germline cysts (Cox and Spradling, 2009), we first checked whether this perinuclear pattern corresponds to mitochondria. Indeed, we observed a small amount of overlap between immunostained mitochondria and Clu:GFP particles (Fig. 2B,F). The early (Rab5:YFP) or late (Rab7:YFP) endosome markers did not colocalize with Clu and expression of a dominant-negative version of Rab5 in the musculature did not result in perinuclear αPS2 accumulation (supplementary material Fig. S1). These results rule out the role of endocytosis as an explanation for the defects in *clu* mutants.

As αPS2 accumulates around the nucleus in *clu* mutants, the distinct accumulation of Clu at the same location favors the idea that Clu may be required for αPS2 trafficking. We next analyzed the subcellular distribution of Clu in larval muscle tissue with respect to the organelles of the early secretory pathway, the ER, ERES, and Golgi. As in vertebrate muscle fibers (Percival and Froehner, 2007; Ralston et al., 2001), we found that the ER pervades the entire cell, including the sarcomere and the nuclear envelope that is continuous with the ER. Approximately 35% (see Materials and Methods for details on quantification) of the KDEL-YFP ER marker was found to co-localize with Clu adjacent to the nuclear envelope (Fig. 2C,F) showing that a portion of Clu localizes to this organelle. The Clu pattern sometimes appears as puncta that could correspond to ERES and we used Sec16 as a marker (Ivan et al., 2008). About 40% of perinuclear ERES marked by Sec16 also contained Clu (Fig. 2D,F). In *Drosophila*, the Golgi apparatus comprises stacked elements that are found in very close proximity to ERES to form tER-Golgi units (Kondylis and Rabouille, 2009). Accordingly, some Clu protein was also detected in the same location as Golgi-YFP puncta that surround the nucleus (Fig. 2E,F). Knockdown of Clu using RNAi also resulted in the retention of αPS2 (supplementary material Fig. S1). Taken together, these data show that Clu is broadly localized within the muscle cell, but a large pool of Clu localizes to the peripheral ER, and co-localizes with ERES and the Golgi to a smaller extent. This localization is consistent with a role in αPS2 export for the ER and transport in the early secretory pathway in muscle cells.

### The role for Clu in mitochondrial distribution is independent of its role in αPS2 localization

**Fig. 3.**
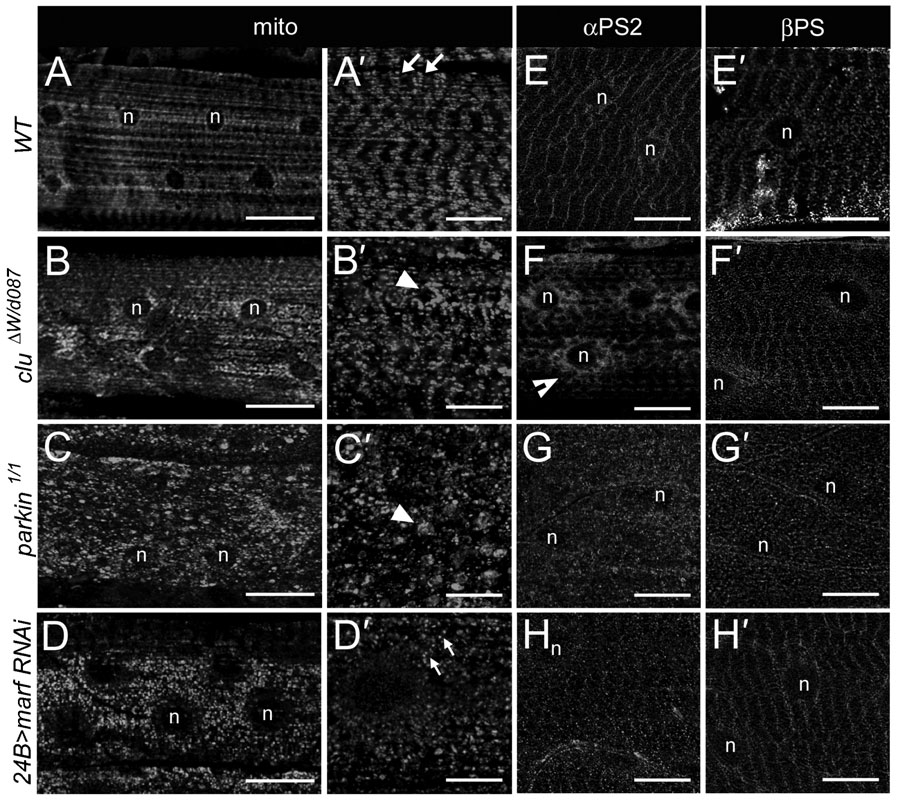
The requirement for Clu and dGRASP in integrin localization is separable from mitochondrial organization. (A–D) The distribution of mitochondria (anti-Complex V) in muscle 6 (n = nucleus). (A–D) Z-stacks of the muscle surface and internal myofibrils. (A′–D′) Internal muscle cell slices. (A,A′) The mitochondria in WT muscle cells are evenly distributed and align in a repeated pattern (arrows). (B–D′) Either *clu*^−*/*−^ (B,B′) or *parkin*^−*/−*^ (C,C′) mutants exhibit severe mitochondrial clustering (arrowheads). Knockdown of *marf RNAi* in the muscle with *24B*-GAL4 results in fragmented mitochondria (small arrows). (E–H) The perinuclear αPS2 localization phenotype is only apparent in *clu* mutants (indented arrows in F), and not upon a decrease in *parkin* (G) or *marf* (H). (E′–H′) βPS does not accumulate in the perinuclear region in WT (E′), *clu*^−*/*−^ (F′) mutants, *parkin*^−*/*−^ mutants (G′), or *marf RNAi* muscles (H′). Scale bars, 50 µm (A–D,G), 10 µm (E–H, A′–H′).

Due to the known role for Clu in mitochondrial dispersion in diverse organisms (Cox and Spradling, 2009; Dimmer et al., 2002; Fields et al., 1998; Zhu et al., 1997), we therefore tested if Clu is required for mitochondrial distribution in larval muscle tissue. In WT myofibers, mitochondria were abundant between adjacent nuclei at the muscle surface muscle (Fig. 3A) and in a repeated sarcomeric pattern within muscles (Fig. 3A′). As expected, the pattern of mitochondrial distribution was severely disrupted in *clu*^−*/*−^ mutant muscles where we observed clustering of mitochondria (Fig. 3B,B′). We next examined if mutants that affect mitochondrial integrity or dynamics phenocopy the *clu* mutant perinuclear accumulation of αPS2 (Fig. 3F). Examination of *parkin*^−*/*−^ mutant muscles revealed multiple mitochondrial aggregates within the cell (Fig. 3C,C′), but no obvious accumulation of intracellular αPS2 around the nuclei (Fig. 3G). To test this further, we knocked down the mitochondrial fusion protein Mitofusin, encoded by the *marf* gene, using RNAi. As expected, we observed a strong mitochondrial fission phenotype (Fig. 3D,D′) in agreement with the published role of Marf (Ziviani et al., 2010). However, *marf RNAi* did not affect αPS2 localization within contractile muscles (Fig. 3H). In all genotypes examined (Fig. 3E′–H′), βPS did not accumulate around muscle nuclei. Thus, we can conclude that within muscle tissue, Clu exhibits two separable roles, one implicated in mitochondrial distribution and the other, to mediate αPS2 transport.

### *dgrasp RNAi* knockdown phenocopies muscle defects upon loss of Clu

**Fig. 4.**
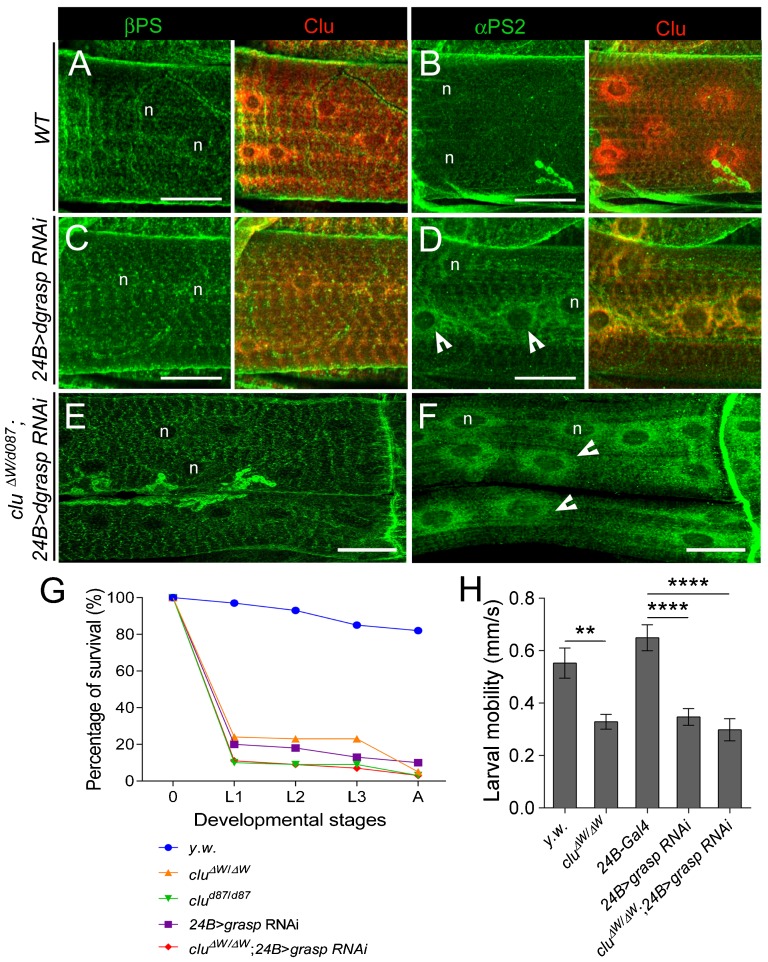
*dGRASP RNAi* in the muscle phenocopies *clu* mutants. (A–D) L3 muscle fillets reveal the localization of integrins (green) and Clu (red). βPS (A) and αPS2 (B) show relatively normal integrin distribution in WT muscle (n = nucleus). In *24B>dgrasp RNAi* muscles, βPS integrin appears WT (C), while the αPS2 subunit colocalizes with endogenous Clu in the nuclear periphery (D; indented arrowheads). (E,F) βPS localization in muscles mutant for *clu* that also knockdown dGRASP levels (*clu^ΔW^/clu^d087^; 24B> dgrasp RNAi)* are similar to WT (E), while the perinuclear distribution of αPS2 looks like *clu*^−*/*−^ or *dgrasp RNAi* alone (F; indented arrowheads). (G) Survival curve for *clu* and *dgrasp* mutants at different developmental stages (E, embryo; L1, 1^st^ instar larva; L2, 2^nd^ instar larva; 3^rd^ instar larva; A, adult). (H) Locomotor activity analysis for early L3 larvae of indicated genotypes (mean±s.e.m.; **p<0.005; ****p<0.0001). Scale bars, 25 µm (A–D); 50 µm (E,F).

The integrin subunit retention phenotype in the perinuclear ER is reminiscent to loss of dGRASP function in the follicular epithelium (Schotman et al., 2008). To directly test if dGRASP functions like Clu in αPS2 delivery in the muscle, we examined the distribution of βPS and αPS2 upon *dgrasp* loss of function. Previously published *dgrasp* mutants were no longer available (Schotman et al., 2008), so we utilized RNAi techniques to knockdown dGRASP function specifically in the L3 musculature. First, we confirmed knockdown of dGRASP protein by examining the intensity of immunofluorescence in *24B>dgrasp RNAi* larval muscles (Fig. 6K; supplementary material Fig. S2). Second, in *dgrasp RNAi* L3 myofibers, βPS localization was WT (Fig. 4A,C), whereas the normal distribution of αPS2 in WT muscles (Fig. 4B) was altered and phenocopied the perinuclear ER localization in *clu* mutant larvae (Fig. 4D). Therefore, Clu and dGRASP loss of function leads to the same αPS2 accumulation in the perinuclear ER.

### *clu* and *dgrasp* function in the same genetic pathway

Since *clu* mutants show an accumulation of αPS2 in L3 muscles similar to *dgrasp RNAi* mutants, we explored whether *dgrasp* and *clu* function in the same genetic pathway to mediate αPS2 trafficking. Indeed, the perinuclear accumulation of αPS2 in *dgrasp* mutants co-localized with the endogenous location of Clu protein (Fig. 4D). To test this further, we performed epistasis experiments. If *clu* and *dgrasp* function in different pathways, double mutants would be expected to exhibit stronger defects than *clu* or *dgrasp* single mutants. If these two genes function together in the same pathway, phenotypes observed in *clu*, *dgrasp* double mutants should be similar to those observed in either mutant alone. Larvae in which both *clu* and *dgrasp* function were simultaneously removed showed αPS2 perinuclear accumulation phenotypes (Fig. 4F), lethality curves (Fig. 4G), and larval locomotion phenotypes (Fig. 4H), nearly identical to those observed in either *clu* or *dgrasp* single mutants alone. These data suggest that Clu and dGRASP are likely to act in the same genetic pathway.

### Clu and dGRASP physically interact

**Fig. 5.**
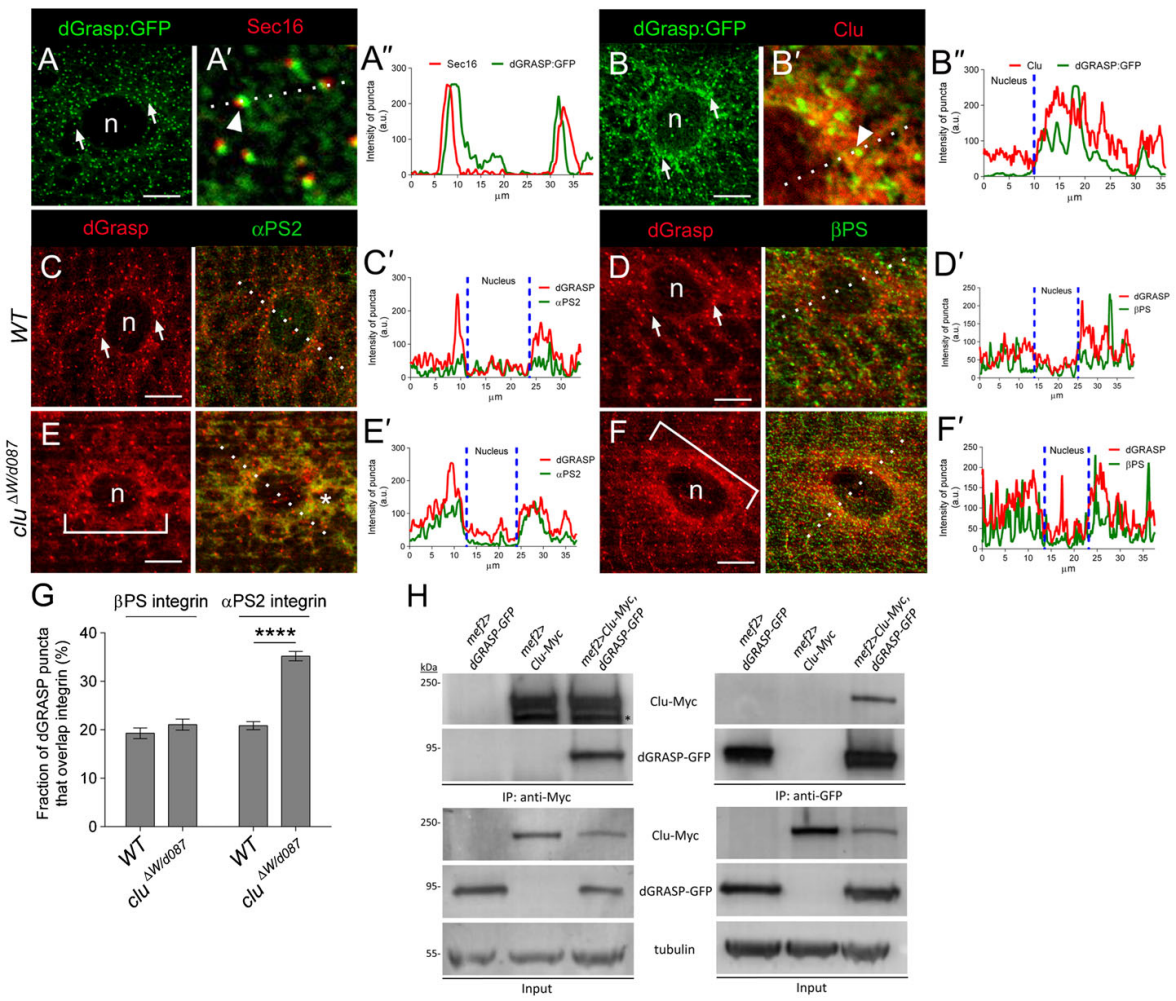
Clu physically binds to and mediates the localization of dGRASP to puncta. (A–B″) UAS-dGRASP-GFP is expressed in the muscle using by *mef2*-GAL4 and is found in puncta surrounding the nuclei (n; arrows in A,B). High magnification images and line intensity profiles (dotted lines) reveal a partial overlap with Sec16 (arrowheads in A′,A″) and colocalization with Clu (arrowheads in B′,B″). (C–F′) Micrographs (C–F) and the corresponding fluorescent intensity line profiles to illustrate colocalization (C′–F′; dotted lines) between dGRASP (red) and integrins (green). The dGRASP-positive puncta (arrows) at the ERES and Golgi exhibit little overlap with αPS2 (C,C′) and βPS (D,D′) in the cell. dGRASP protein is more diffuse in *clu* mutants (E,F; brackets) and colocalizes with αPS2 around the nuclei (asterisk in E,E′). (G) Quantitation of results in panels C–F showing the fraction of dGRASP signal that colocalizes with integrins (mean±s.e.m.; ****p<0.0001). (H) A myc-tagged version of Clu and a dGRASP-GFP fusion protein were expressed using the GAL4/UAS system in the L3 stage. Immunoprecipitation of the resulting lysates with either anti-myc (left panel) or anti-GFP (right panel) resulted in the detection of a Clu-dGRASP complex using Western blot analysis. Asterisk indicates background band. Scale bars, 5 µm (A–F).

To gain evidence that the above genetic interaction reflects a functional role for a Clu-dGRASP complex in αPS2 transport, we first determined whether Clu and dGRASP colocalized in muscle tissue. In yeast and *Drosophila*, dGRASP is known to localize to both the ERES and Golgi on what is termed a transitional ER (tER)-Golgi unit (Behnia et al., 2007; Kondylis et al., 2005; Vinke et al., 2011). We expressed UAS-*dGRASP-GFP* in WT muscle cells using *mef2*-GAL4. Both dGRASP:GFP (Fig. 5A) and endogenous dGRASP protein (supplementary material Fig. S2) are enriched around the nucleus in a punctate pattern consistent with a Golgi localization (supplementary material Fig. S2). Furthermore, dGRASP shows a close proximity to Sec16 (Fig. 5A′,A″; supplementary material Fig. S2), suggesting that, as in other systems, dGRASP localizes to both Golgi and ERES. This is consistent with Clu localization (Fig. 2). To confirm this, we double labeled dGRASP:GFP and Clu and found a partial but significant overlap around the nuclei (Fig. 5B–B″). In the reverse experiment, we also found that ∼50% of the endogenous Clu:GFP fusion protein colocalizes with dGRASP protein detected using anti-dGRASP antisera (supplementary material Fig. S2).

We next assessed if Clu or dGRASP are reliant on one another for their perinuclear localization. Indeed, we found that the WT dGRASP localization pattern was altered upon loss of Clu. dGRASP appeared more diffuse in the cytoplasm (Fig. 5E,F, brackets) when compared to the tight localization in WT puncta (Fig. 5C,D), suggesting that Clu is involved in dGRASP localization and in the organization/dispersion of the early secretory pathway. The dGRASP clustering around the nucleus was reminiscent of the αPS2 pattern also observed in the *clu* mutant. Accordingly, αPS2 and dGRASP showed tight colocalization (Fig. 5E–E″,G) whereas βPS was less affected (Fig. 5F–F″,G). Of note, Clu localization was unaffected in *dgrasp RNAi* muscle cells (data not shown).

The dependence of dGRASP on Clu for WT localization, as well as the strong similarity in the αPS2 phenotype upon loss of function of both proteins, suggests the possibility that dGRASP and Clu may form a physical interaction. To test this hypothesis, we performed co-immunoprecipitations (co-IPs) of tagged forms of Clu and dGRASP from L3 larval lysates (Fig. 5I). Immuno-isolation of dGRASP-GFP using anti-GFP beads resulted in the detection of Clu in a biochemical complex by Western blotting. In the reciprocal experiment, we were able to detect dGRASP in IPed Clu-myc complexes. However, we were not able to detect a Clu-dGRASP complex in control lysates that did not have tagged forms of both Clu and dGRASP together. Our results strongly suggest that a Clu-dGRASP biochemical complex is required for αPS2 export from the perinuclear ER where it is synthesized from its targeted mRNA followed by its transport to the plasma membrane.

### Sec16 stability depends on Clu

As mentioned above, transport out of the ER typically occurs at ERES and one of the key proteins in ERES functional organization is Sec16. To test whether this is also true for integrin subunits, we depleted Sec16 in the muscle and examined whether integrin localization was changed. First we confirmed that Sec16 protein levels were reduced upon *sec16 RNAi* knockdown in muscle tissue (supplementary material Fig. S3). Next, we found that loss of Sec16 altered the normal localization of αPS2 (Fig. 6A), where it was found strongly concentrated around the perinuclear ER (Fig. 6B,E), in agreement with its site of synthesis (Fig. 1I). We also confirmed this perinuclear accumulation of αPS2 protein by expressing a second, independently generated *sec16 RNAi* construct (supplementary material Fig. S3). This suggests that as expected, αPS2 uses ERES machinery for ER exit. Accordingly, there was also a small increase in βPS perinuclear accumulation (Fig. 6D,E), as might be expected for the depletion of any components of the ERES.

To examine how Clu or dGRASP may alter integrin transport out of the ER via ERES, we examined the distribution and levels of Sec16 protein in mutant backgrounds. Sec16 levels in *clu* mutants was reduced, shown both by IF (Fig. 6G) and WB (Fig. 6K). The number (Fig. 6I) and relative size (Fig. 6J) of Sec16 puncta were smaller compared to WT (Fig. 6F). This result was specific for *clu* mutants, as Sec16 protein levels were not altered in *dgrasp RNAi* muscle cells (Fig. 6H–K). Altogether, we propose a model in which Clu forms a complex with dGRASP to maintain its localization in the perinuclear early secretory pathway as well as maintaining Sec16 stability. This, in turn, is necessary for the ERES function and αPS2 exit out of the ERES.

### ER stress induced by loss of Clu or dGRASP is ameliorated by chemical chaperones

**Fig. 7.**
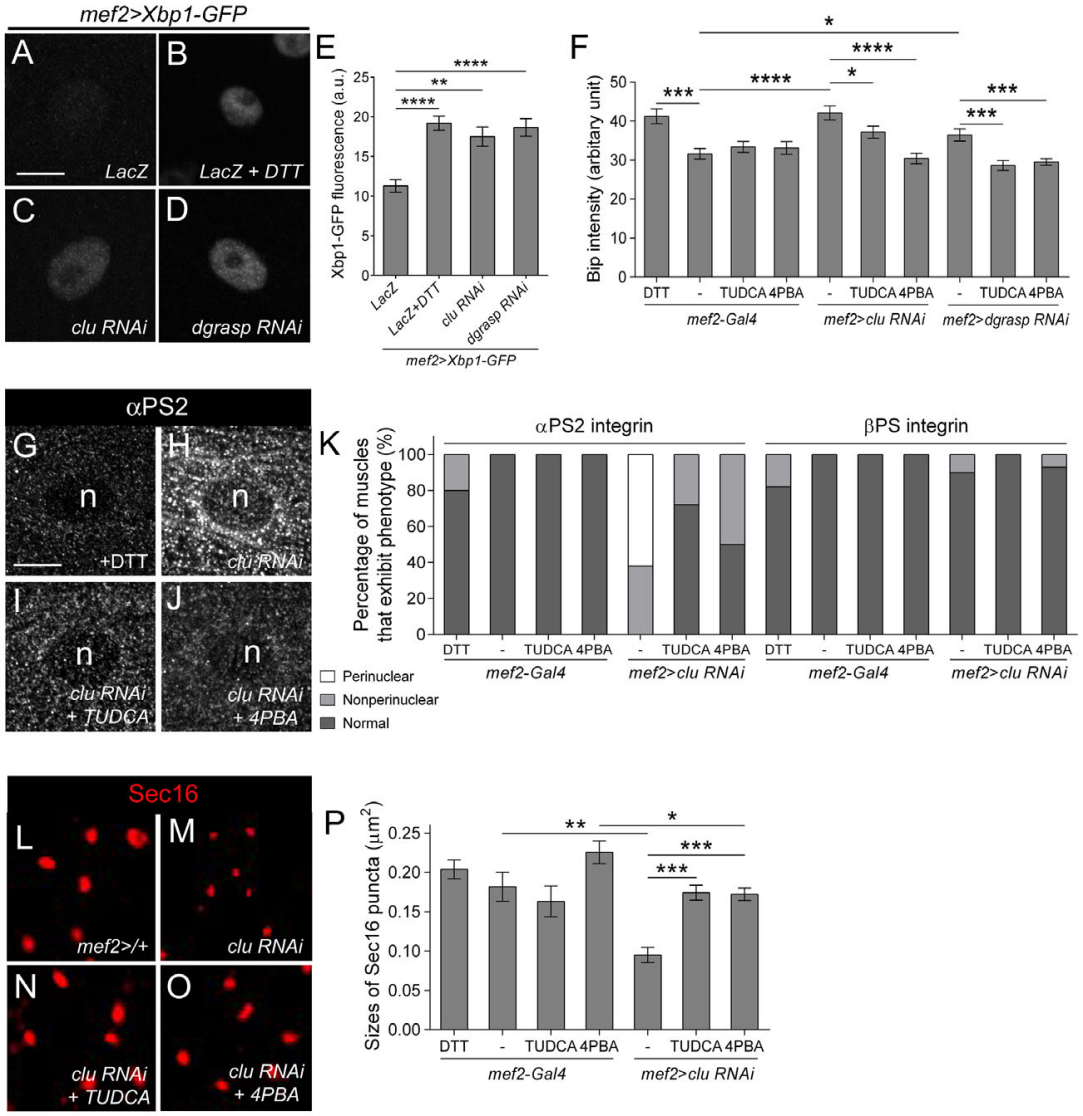
Molecular chaperones can alleviate ER stress due to a reduction in either Clu or dGRASP. (A–F) ER stress markers are upregulated in *clu* or *dGRASP RNAi*. The ER stress reporter Xbp1-GFP is elevated upon induction of ER stress by DTT (B) or upon RNAi knockdown of *clu* (C) or *dGRASP* (D) in L3 muscles (n = nucleus). (E) Quantitation of the ER stress inducer Xbp1-GFP in the indicated genotypes. (F) Independent measurements of ER stress measuring the amount of Bip levels in L3 muscle. ER stress in increased upon feeding with DTT or in *clu* or *dGRASP RNAi* and can be ameliorated upon treatment with the molecular chaperones TUDCA or 4PBA (*p<0.05; ***p<0.0005; ****p<0.0001). (G–K) αPS2 accumulates in *clu RNAi* (H) muscle tissue and this perinuclear accumulation is alleviated upon treatment with TUDCA (I) or 4PBA (J). (K) Graph depicting the internal accumulation of αPS2 upon loss of Clu only. (L–P) The size of Sec16 ERES is reduced in *clu RNAi* (M), but is restored upon inhibition of ER stress (N–P) (mean±s.e.m.; *p<0.05; **p<0.01; ***p<0.005). Scale bars, 10 µm (A–D, G–J); 2 µm (L–O).

Protein stability and localization can be affected by several stresses (Barlowe and Miller, 2013), and given Sec16 localization to ERES, we asked whether Sec16 decline in *clu* mutants could be a consequence of ER stress. In response to the accumulation of misfolded or unfolded proteins in the ER, one such marker for ER stress is the transcription factor Xbp1 (Sone et al., 2013). In L3 contractile muscles, oral intake of the ER stress inducer DTT resulted in upregulation of the XBP1-GFP reporter (Fig. 7B,E) when compared to non-DTT fed control larvae (Fig. 7A,E). RNAi knockdown of *clu* (Fig. 7C) or *dgrasp* (Fig. 7D) also induced activation of the Xbp1-GFP reporter (Fig. 7E). We confirmed and extended these results using the ER stress marker Binding immunoglobulin protein (BiP). As expected, there was an increase in BiP immunostaining (supplementary material Fig. S4) upon exposure of L3 muscles to DTT or in *clu* and *dgrasp RNAi* muscles (Fig. 7F). Importantly, we found that both ER stress (Fig. 7F) and αPS2 accumulation (Fig. 7G–K) were reduced upon treatment with the chemical chaperones tauroursodeoxycholic acid (TUDCA) and 4-phenylbutyric acid (PBA), which both relieve ER stress (Oslowski and Urano, 2011; Samali et al., 2010). Furthermore, amelioration of ER stress also rescued the size of Sec16-positive puncta, or size of ER exit sites upon a reduction in Clu (Fig. 7L–P). We conclude that αPS2 accumulates in the ER as a result of ER stress induced upon loss of Clu and dGRASP.

## DISCUSSION

Our data demonstrate a novel role for Clu in αPS2 exit from the perinuclear ER in larval muscle that is different from previously reported roles. As mentioned previously, the first established function is in the prevention of mitochondrial clustering (Cox and Spradling, 2009; Dimmer et al., 2002; Fields et al., 1998; Zhu et al., 1997). The second role of Clu regulates aPKC activity in neuroblast stem cell divisions (Goh et al., 2013). A third role for Clu was published just before submission of this manuscript. Mammalian CLUH can function as an mRNA-binding protein for RNAs encoding nuclear mitochondrial proteins, thus possibly providing a link for mitochondrial biogenesis and localization (Gao et al., 2014). Thus, Clu is a multifaceted protein whose cellular and developmental roles are just beginning to be elucidated.

### The role of the Clu-dGRASP complex in αPS2 ER exit

Here we show that αPS2 is synthesized from a pool of mRNA that is targeted around the nucleus. As αPS2 is a transmembrane protein, this would allow for local synthesis of this protein in the perinuclear ER. This same idea has been proposed in polarized cells, where the coupling of mRNA retention and local translational allows for efficient sorting to the final sites of membrane deposition and/or secretion (Herpers and Rabouille, 2004). When the machinery for αPS2 ER exit is disrupted, αPS2 is retained in the perinuclear ER, as observed in Clu and dGRASP. How *αPS2* mRNA is targeted to this location is not known. The ER can form either networked tubules or stacked sheets, the latter being more abundant around nuclei (Joensuu et al., 2014; Terasaki et al., 2013) and it is therefore possible that ER structure plays a role in mRNA targeting.

Both Clu and dGRASP form a complex that functionally localizes to ERES. The role of this complex could be either direct, such as an interaction with ER cargo receptors such as p24 family members (Strating and Martens, 2009), or indirect. For instance, loss of Clu or dGRASP could affect the microtubule (MT) network and compromise the functional integrity of ERES. Previous data shows that the MT cytoskeleton is closely associated with the reorganization of tER-Golgi units near the nuclear envelope in rat contractile myofibers (Ralston et al., 2001). However, we were able to rule out a role for the MT cytoskeleton in αPS2 delivery. Loss of Clu or dGRASP did not alter the organization of the MT network in larval muscle cells. Furthermore, disruption of the MT cytoskeleton by muscle-specific overexpression of the MT-severing protein Spastin (Sherwood et al., 2004) in L3 larval muscles did not recapitulate the perinuclear accumulation of αPS2 (data not shown).

Clu acts to mediate αPS2 export through modulation of Sec16 stability, a key factor required for COPII coated vesicle dynamics. We also show that Clu and dGRASP act to inhibit ER stress. Upon loss of Clu, ER stress increases, leading to Sec16 degradation and impairment of αPS2 export, and ER retention. Importantly, alleviating ER stress with the chemical chaperones TUDCA and 4PBA suppressed both αPS2 accumulation and the size of ERES. This data provides at least one mechanism for the regulation of αPS2 transport by Clu-dGRASP in myofibers.

### ER stress and mechanical stress

The biological inputs that trigger ER stress in muscle tissue are not clear. Studies in *Drosophila* follicle cells support the intriguing hypothesis that integrins trigger their own mode of transport in response to mechanical stress (Schotman et al., 2009). The physical tension generated during epithelial remodeling induces an upregulation of *dgrasp* mRNA and is dependent upon integrins and the subsequent recruitment and/or activation of RhoA and the LIM protein PINCH (Schotman et al., 2009). Interestingly, elevated PINCH levels also suppress hypercontraction muscle mutants (Pronovost et al., 2013). Thus, maybe PINCH is a key sensory component in tissues that sense, transduce, and alter secretion routes of proteins to withstand changes in physical forces. Supporting this idea are multiple pieces of evidence where changes in patterned muscle activity alter the distribution of the Golgi and ERES (Jasmin et al., 1989; Percival and Froehner, 2007; Ralston et al., 2001). Furthermore, The RNA binding protein HOW is involved in *dgrasp* mRNA stability the in the follicular epithelium (Giuliani et al., 2014) and interesting, *how* mutants show a muscle phenotype (Baehrecke, 1997; Nabel-Rosen et al., 1999). If Clu is acting as a sensor in transducing mechanical stress, for example, it may have the ability to alter the trafficking of proteins in response to such physiological changes.

### Classical secretion of integrins versus Golgi bypass in muscle cells

The general organization of ERES and the Golgi complex seem conserved between *Drosophila* and mammalian skeletal muscles, where these organelles are broadly distributed throughout the cell with accumulation around nuclei (Percival and Froehner, 2007; Ralston et al., 2001). Studies of glycoprotein processing show that multiple delivery routes exist in multinucleated myotubes (Rahkila et al., 1998). For example, influenza virus hemagglutinin (HA) is transported through the Golgi to the cell surface in rat L6 muscle cells. However, half of the pool of labeled vesicular stomatitis virus (VSV) G protein exits the ER but gets shuttled into intracellular vesicles independent of the Golgi. It is not surprising that the complexity of muscle cells may require multiple or redundant routes for membrane delivery.

Like αPS2 in our system, the α integrin subunit (αPS1) in the *Drosophila* follicular epithelium is also retained in the ER in the absence of dGRASP function and reaches the plasma membrane in a Golgi independent manner (Schotman et al., 2008). This leads to the question as to whether αPS2 in larval muscles also bypasses the Golgi. Our preliminary results of Syntaxin 5 (an essential SNAREs for protein transport to and through the Golgi) knockdown showed severely impaired larval survival, but did not phenocopy the *clu* or *dgrasp* αPS2 accumulation phenotype (data not shown). This suggests that αPS2 could bypass the Golgi. However, biochemical evidence demonstrating the presence or absence of Golgi-specific post translational modifications have proven difficult to gather and it remains an open question. Interestingly, in HeLa cells, Golgi bypass of CFTR has been linked to ER stress leading to GRASP55 binding to the C-terminal PDZ1 domain of CFTR (Gee et al., 2011).

One outcome from this work is a departure from the notion that α/β heterodimer formation is a prerequisite for ER exit, and therefore the accumulation of αPS2, but not βPS is counterintuitive. Of note, βPS is not excluded from the perinuclear ER, so the role of Clu as a chaperone might still hold true. Nevertheless the ER export of integrins (as a complex or as individual subunits), at least in *Drosophila*, might be more complex than anticipated and might change at different stages of development. Taken together, require more studies to determine what domains of Clu and/or interacting partners are essential for various cellular activities.

## Supplementary Material

Supplementary Material
