# Supplementary material for: Loss of a Clueless-dGRASP complex results in ER stress and blocks Integrin exit from the perinuclear endoplasmic reticulum in Drosophila larval muscle
Source: Biol Open. 2015 Apr 10;4(5):636–48. doi: 10.1242/bio.201511551 (PMC4434815; doi:10.1242/bio.201511551)
Supplement: Supplementary Material [file supp_4_5_636__index.html]

Loss of a Clueless-dGRASP complex results in ER stress and blocks Integrin exit from the perinuclear endoplasmic reticulum in Drosophila larval muscle — Loss of a Clueless-dGRASP complex results in ER stress and blocks Integrin exit from the perinuclear endoplasmic reticulum in Drosophila larval muscle — Supplementary Material 

# Loss of a Clueless-dGRASP complex results in ER stress and blocks Integrin exit from the perinuclear endoplasmic reticulum in *Drosophila* larval muscle

## bio.201511551 Supplementary Material

**Files in this Data Supplement:**

- Supplementary Material - Zong-Heng Wang et al. doi: 10.1242/bio.201511551
