# Supplementary material for: Loss of a Clueless-dGRASP complex results in ER stress and blocks Integrin exit from the perinuclear endoplasmic reticulum in Drosophila larval muscle
Source: Biol Open. 2015 Apr 10;4(5):636–48. doi: 10.1242/bio.201511551 (PMC4434815; doi:10.1242/bio.201511551)
Supplement: Supplementary Material [file supp_bio.201511551_bio.201511551-s1.pdf]

 Biology Open (2015) 000, 1–13 doi:10.1242/bio.201511551

Supplementary Material

Zong-Heng Wang et al. doi: 10.1242/bio.201511551

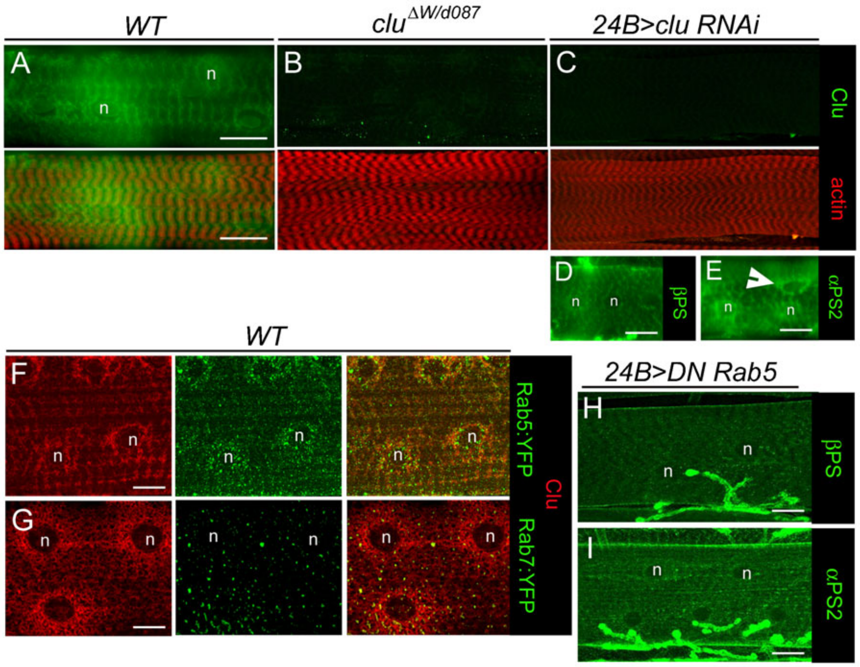

Fig. S1. Specificity of Clu antibody and Clu is not required for endocytosis of integrins. (A–C) Clu (green) is present around the nuclei (n) and is associated with the Z-line in WT L3 muscles (A). This staining is greatly reduced in *clu* mutants (B) or muscle-specific knockdown of *clu RNAi* using *24B*-GAL4 (C). (D,E) *24B>clu RNAi* animals recapitulate the αPS2 retention phenotype (indented arrow in E), while βPS (D) is not affected. (F,G) Clu protein do not appreciably colocalize with the early endosome Rab5:GFP (F) or the late endosome Rab7:GFP (G) markers. (H,I) Expression of a dominant-negative form of Rab5 in the L3 musculature did not affect βPS (H) or αPS2 (I) localization. Scale bars, 20 µm (A–E,H,I); 10 µm (F,G).

Biology Open

Biology Open (2015) 000, 1–13 doi:10.1242/bio.201511551

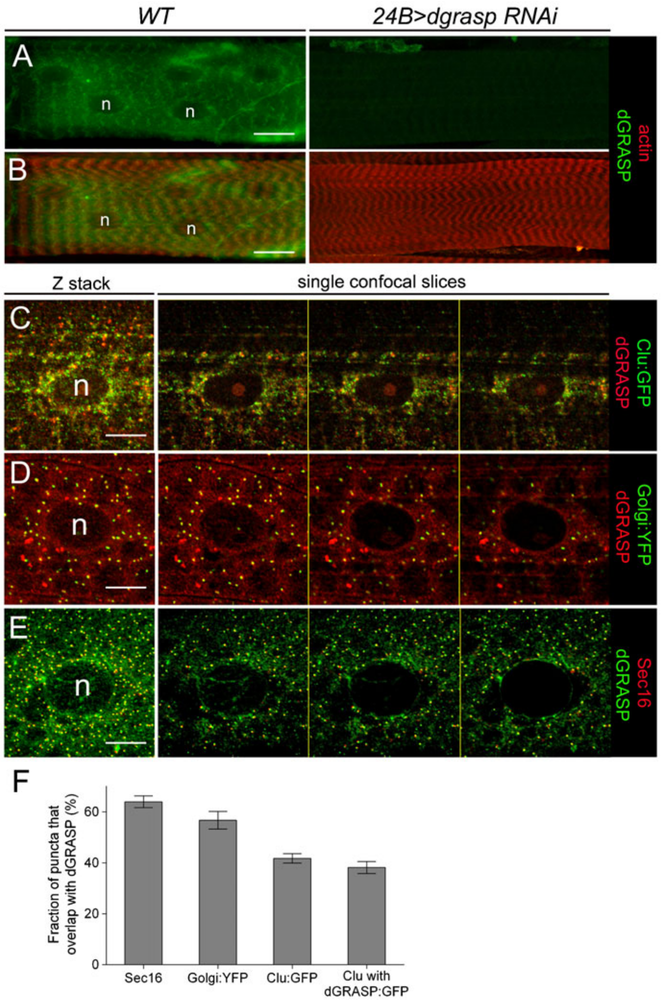

Fig. S2. dGRASP localizes to ERES. (A,B) The normal localization of anti-dGRASP immunolocalization (green) is largely absent upon *dGRASP RNAi* knockdown in muscle tissue. (C–F) Colocalization of dGRASP with organelle markers. (C) dGRASP is observed at the same location as Clu:GFP. (D,E) dGRASP protein colocalizes with both Golgi (D) and ERES as marked by Sec16 protein (E). (F) Quantification of dGRASP colocalization with organelle markers around the nuclei (n). Analysis was conducted using single confocal planes. Mean±s.e.m. Scale bars, 20 µm (A,B); 10 µm (C–E).

Biology Open (2015) 000, 1–13 doi:10.1242/bio.201511551

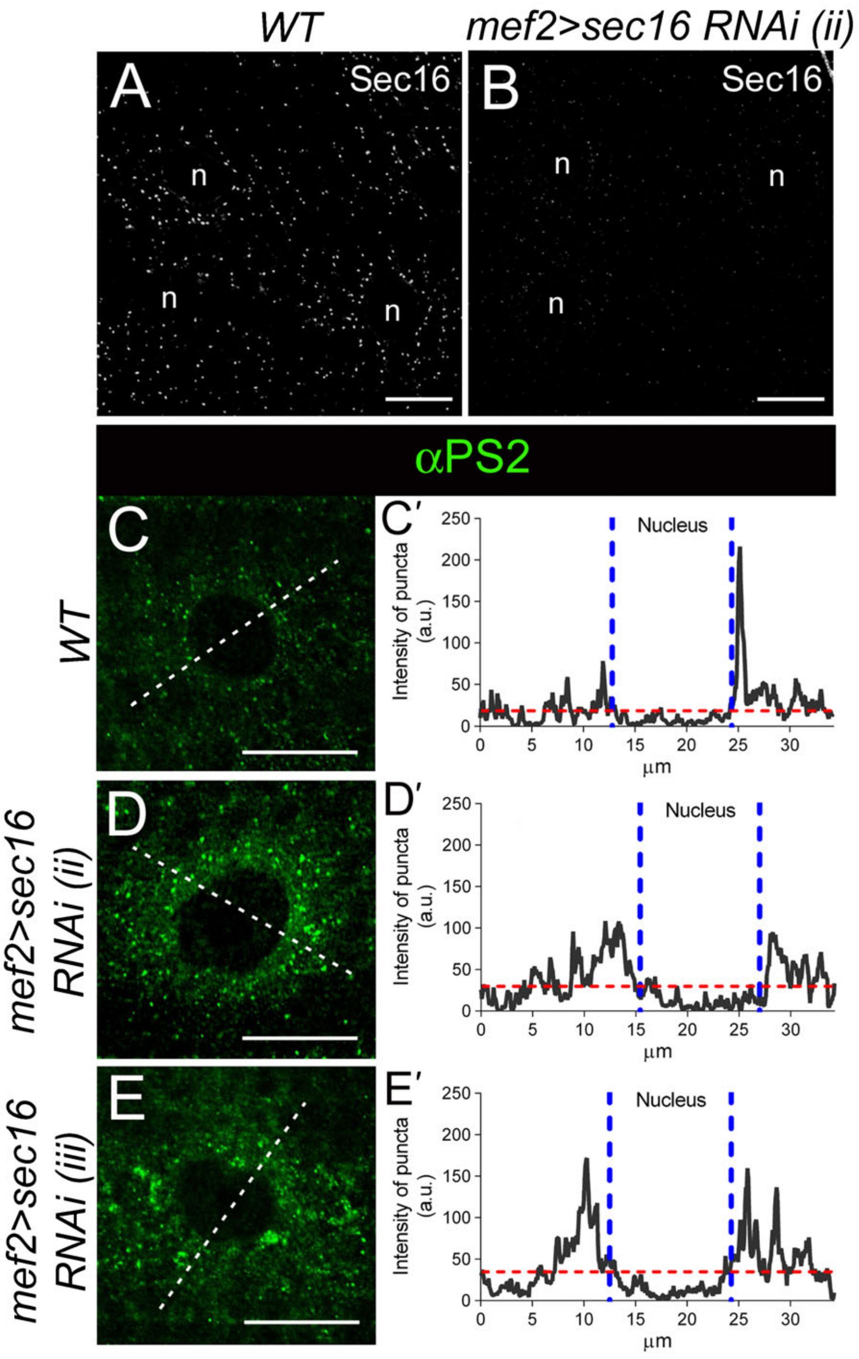

Fig. S3. Sec16 protein levels are reduced in *sec16 RNAi* muscle tissue. (A,B) Staining of ERESs by anti-Sec16 antisera around the nuclei (n) in WT L3 larval muscle (A) is reduced upon expression of *sec16 RNAi* (on chromosome II) using *mef2*-GAL4 (B). (C–E) αPS2 integrin staining in WT (C) or upon Sec16 knockdown using independent *UAS-sec16 RNAi* constructs inserted on chromosomes 2 (D) or 3 (E). (C′–E′) Plot profiles (dotted lines) in panels C–E show the intensity of αPS2 perinuclear accumulation across nuclei. Note that the intensity (vertical peaks) and distribution (span of horizontal peaks) of αPS2 is greater in *sec16 RNAi* (D′,E′) than in WT (C′). The dashed red lines indicate the average background pixel intensity from multiple images. Scale bars, 10 µm (A–E).

Biology Open

Biology Open (2015) 000, 1–13 doi:10.1242/bio.201511551

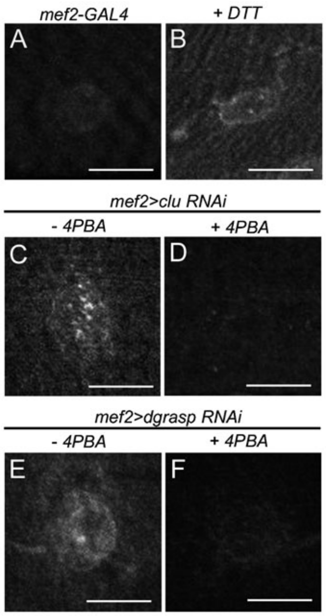

Fig. S4. The ER stress reporter BiP is increased upon loss of Clu or dGRASP and decreased upon amelioration of ER stress.
(A–F) Perinuclear staining of BiP protein in the indicated genotypes. (A) The control *mef2*-GAL4 shows little BiP staining. ER stress, as marked by BiP staining, is increased by the addition of DTT (B), or a decrease in Clu (C) or dGRASP (E). This staining is again decreased upon the addition of the ER stress reliever, 4PBA. Scale bars, 10 µm (A–F).
